# Adaptive immune responses to SARS-CoV-2 in DMARD-treated patients with chronic inflammatory rheumatisms

**DOI:** 10.1136/rmdopen-2025-005673

**Published:** 2025-07-05

**Authors:** Maxime Beretta, Emmanuel Martin, Olivier Fogel, Clementina López-Medina, Cyril Planchais, Thomas Bruneau, Pedro Goncalves, Jerome Avouac, Francis Berenbaum, Jérémie Sellam, Bruno Fautrel, Jacques Morel, Beatrice Parfait, James P Di Santo, Sylvie Behillil, Sylvie van der Werf, Helene Péré, Sylvain Latour, Hugo Mouquet, Corinne Miceli-Richard

**Affiliations:** 1 Humoral Immunology Unit - INSERM U1222, Institut Pasteur and Universtity Paris Cité, Paris, France; 2 Laboratory of Lymphocyte Activation and Susceptibility to EBV infection, INSERM UMR 1163, Imagine Institute, Paris, France; 3 Department of Rheumatology, Hôpital Cochin - Assistance Publique Hôpitaux de Paris - Université Paris Cité, Paris, France; 4 Rheumatology, Reina Sofia University Hospital, Cordoba, Spain; 5 Service de Microbiologie (Unité de virologie), Hôpital Européen Georges Pompidou, Paris, France; 6 Inserm U1223, Innate Immunity Unit, Institut Pasteur, Paris, France; 7 Faculty of Medicine Pierre & Marie Curie Paris VI, Hopital Saint-Antoine, Paris, France; 8 Immunorhumatologie, CHU Lapeyronie, Montpellier, France; 9 Centre de Ressources Biologique - Hôpital Cochin - Fédération des Centres de Ressources Biologiques-, Assistance Publique Hôpitaux de Paris - Université Paris Cité, Paris, France; 10 UMR 3569 CNRS, Molecular Genetics of RNA Viruses, National Reference Center Respiratory Viruses, Institut Pasteur, Paris, France; 11 Immunoregulation Unit, Institut Pasteur, Paris, France

**Keywords:** COVID-19, B-Lymphocytes, Antirheumatic Agents, DMARD, T-Lymphocytes

## Abstract

**Background:**

Patients with rheumatoid arthritis (RA) and spondyloarthritis (SpA) are at an increased risk for infection related to the use of immunomodulatory therapies (ITs). The objective of this study is to assess the impact of ITs on the adaptive immune responses to SARS-CoV-2.

**Methods:**

The study population comprised 94 patients (48 SpA; 46 RA; mean age of 53±14 years) with a confirmed SARS-CoV-2 infection. 20 age-matched individuals (50±17 years), corresponding to the patients’ household contacts infected at the same time, were included as the control population. Patients were stratified by treatment groups: methotrexate (MTX)/sulfasalazine (n=17/2), anti-TNF (n=24), anti-TNF+MTX (n=23), RTX (N=11), anti-IL17 (n=7) and others (n=11). The study compared the viral loads in plasma, stools and nasal swabs and the SARS-CoV-2-specific humoral and cellular immune responses (antibodies, B and T lymphocytes) following SARS-CoV-2 infection.

**Results:**

Viral persistence was not observed in the blood, nasopharynx and stools of patients undergoing ITs. Overall, the SARS-CoV-2-specific humoral and T-cell responses were preserved. Patients receiving RTX showed significantly lower IgA and IgG responses to SARS-CoV-2 compared with other treatment groups. Most patients, including RTX recipients, exhibited significant CD4+T cell responses. However, RTX therapy was associated with reduced SARS-CoV-2-specific activated CD8+T cells. A correlation was observed between humoral immune parameters and CD8^+^ T cell activation.

**Conclusions:**

While most patients demonstrated the capacity to mount an immune response to SARS-CoV-2, treatment with RTX impacted both humoral and CD8+cell responses. Developing vaccines that elicit robust CD8+T cell responses could offer benefits to individuals undergoing ITs for inflammatory rheumatic diseases.

**WHAT IS ALREADY KNOWN ON THIS TOPIC**

- Overall, several studies, including those conducted by the COVID-19 Global Rheumatology Alliance, have offered somewhat reassuring perspectives on the progression of COVID-19 in individuals with rheumatic diseases receiving immunomodulatory drugs. However, there is a scarcity of data on the SARS-CoV-2 immune responses among non-vaccinated patients undergoing immunomodulatory treatments, particularly concerning CD4-specific and CD8-specific T cells.

**WHAT THIS STUDY ADDS**

- We noted the absence of viral persistence in the blood, nasopharynx and stools in patients undergoing immunomodulatory drugs. SARS-CoV-2-specific humoral and T-cell responses were generally comparable between the various treatment groups. The majority of patients, including those receiving RTX, showed substantial CD4+T cell responses. However, RTX therapy was associated with diminished SARS-CoV-2-specific activated CD8+T cells (CD137+CD69+), and a significant association was observed between humoral response and CD8+T cell activation.

**HOW THIS STUDY MIGHT AFFECT RESEARCH, PRACTICE OR POLICY**

- Our findings reveal an association between blood SARS-CoV-2-specific humoral and CD8+T cell responses. In the realm of autoimmune diseases, B-CD8 crosstalk has been previously noted, in which B-cell depletion results in a subdued pathogenic CD8 T-cell response. The data presented here suggest that opting for vaccines capable of inducing robust CD8+T cell responses could be advantageous for individuals undergoing immunomodulatory drugs for inflammatory rheumatic diseases, especially for patients treated with B-cell depleting therapies.

## Introduction

 Rheumatoid arthritis (RA) and spondyloarthritis (SpA) are the two most common chronic inflammatory rheumatisms with a prevalence of 0.5%–1% for RA^1^ and ~0.35% for SpA.^2^ Several studies have described an increased risk of serious infectious diseases associated with increased morbidity and mortality.^3^,^5^ This risk is comparable between patients with SpA or RA but varies in patients on biological drugs, between 22 and 34 number of infections per 100 patients per years of follow-up depending on the studies.^6^,^8^ This increased risk—frequency and severity—results from the disease itself, especially if the rheumatism is very active,^9^ but also and mainly from the immunosuppressive treatments used.^10^,^12^ The risk of infection is greater for patients on targeted disease-modifying antirheumatic drugs (DMARDs) compared with those on conventional synthetic DMARDs (csDMARDs—mainly methotrexate (MTX)), and the combination of corticosteroid therapy with biological treatment further increases this risk.^6 8^ Lung and upper airways infections are the most common infections observed with targeted DMARDs.^6^,^8^ The risk of infection may differ according to the considered DMARDs.^12^ In addition, immunosuppressed RA or SpA patients have a significant impairment of vaccine responses.^13^ Regarding COVID-19, patients are more vulnerable to SARS-CoV-2 infection and have an increased mortality rate.^14 15^ Moreover, case studies have reported the emergence of viruses carrying escape mutations in persistently infected immunocompromised hosts, facilitating the emergence of new variants.^16^,^18^ Based on analyses of the largest collection of COVID-19 cases among patients with rheumatic diseases (COVID-19 Global Rheumatology Alliance), a study revealed that patients exposed to higher doses of glucocorticoids (≥10 mg/day) had a higher likelihood of hospitalisation, while those treated with anti-TNF agents were associated with a reduced odds of hospitalisation.^19^ CsDMARDs and biological/targeted synthetic (b/ts) DMARDs were not associated with severe COVID-19, but RA patients treated with rituximab or Janus kinase (JAK) inhibitors at COVID-19 onset had increased COVID-19 severity.^20^ Yet, these studies have provided rather reassuring insights on the course of COVID-19 infection in patients with rheumatic diseases treated with immunomodulatory drugs.

The immune response against SARS-CoV-2 relies on both innate and adaptive immunity, notably specific T cells and antibodies. Antibodies to SARS-CoV-2 recognise the viral proteins including the nucleoprotein (N) and the ‘Spike’ glycoprotein (or protein S) that interacts with the SARS-CoV-2 receptor, the human ACE-2.^21^ SARS-CoV-2 neutralising antibodies target the S protein, and levels of serum anti-S IgG antibodies, particularly those binding to the receptor-binding domain (RBD), are strongly correlated to their neutralising activities.^22^ Anti-SARS-CoV-2 IgG and IgM antibodies become detectable 1–2 weeks following the onset of clinical symptoms, with titres gradually decreasing during ~6 months postinfection (PI).^23^ Still, SARS-CoV-2-specific T cells and B cells appear more stable over time, at least in healthy controls.^24^

Knowledge on the immune responses induced by SARS-CoV-2 infection in non-vaccinated patients undergoing immunosuppressive treatments is scarce. In fact, more information has been collected on immune responses to SARS-CoV-2 vaccination. The COVIRIC study has prospectively assessed the impact of immunosuppressive therapies in patients with chronic inflammatory rheumatic diseases on viral load, SARS-CoV-2-specific on humoral and T-cell immune responses during COVID-19, in comparison to members of their family cluster infected with the same viral strain but not under immunosuppressive treatments. These results may help establish guidelines on the measures to be taken in case of a SARS-CoV-2 infection in non-vaccinated patients. More generally, these data could allow modelling the impact of immunomodulatory treatments on the adaptive immune responses in the context of viral infections.

## Results

### Patient and control characteristics

The first patient first visit was held on 11 June 2020 and the last patient last visit on 16 February 2022. 94 patients treated with csDMARD or bDMARDs or tsDMARDs were included in the COVIRIC study (mean age 53±15 years, 66% women). The main characteristics of the patients are reported in table 1. All RA and SpA patients were classified into six groups according to their treatments: csDMARDs monotherapy (MTX: n=17; sulfasalazine: n=2) (median age of 65±15 years); anti-TNF monotherapy (n=24, median age of 43.5±15 years); anti-TNF combotherapy (n=23, median age of 56±13 years); rituximab (RTX), only one patient with RTX without MTX (n=11, median age of 55±11 years); anti-IL17 treatments (n=7, median age of 54±13 years). Patients treated with other bDMARDs or tsDMARDs were gathered in the ‘other’ treatment group: tocilizumab (n=5); JAKi (n=3); abatacept/ABA (n=2) (median age of 62±17 years). Control samples were obtained from the blood of three groups of volunteer healthy donors: individuals not exposed to SARS-CoV-2 (N=5, median age of 41±6 years), individuals who were infected with SARS-CoV-2 1–6 months before (N=9, median age of 33±12 years), and individuals infected with SARS-CoV-2 at the time of inclusion (n=20, mean age 49±16 years). This last group comprised individuals living in the same household (spouses, adult children) who were infected at the same time as the patients. Samples from patients and healthy family members were collected during the visits conducted at 1 month (29±13 days; n=21), 3 months (110±23 days; n=101), 6 months (231±35 days; n=50) and 12 months (368±19 days; n=28) PI. A postvaccination (PV) visit was scheduled 6 months after the first booster injection (Astra Zeneca COVID-19 vaccine n=6, BNT162b2—Pfizer/BioNTech or Moderna mRNA-1273 vaccine n=67, combined regimen n=5). Based on the patients' availability for follow-up visits and the volume of blood obtained at each visit, the complete data set including viral persistence and SARS-CoV-2-specific B-cell and T-cell responses was available among 40 patients of the cohort and 18 of their healthy family members. Antibody responses were studied for the 94 patients and 20 healthy family members. Most patients in the COVIRIC cohort did not experience a severe form of COVID-19, as they were eligible for inclusion between 4 and 6 weeks from the onset of infection (D1) and for longitudinal follow-up. Only four patients had COVID-19-related lung involvement with a lung surface area greater than 50% of the lung parenchyma. Among them, two patients had to be hospitalised in the intensive care unit. These patients with a severe form were treated with MTX as monotherapy (10–25 mg/week). Two of them had treated arterial hypertension, diabetes and a history of myocardial infarction, one had isolated obesity (body mass index 36) and the last had no specific risk factors for the severity of a COVID-19 infection.

**Table 1:** Patients and controls characteristics

	RA (n=46)	SpA (n=48)	Controls (N=20)
Female, n (%)	41 (89)	22 (45)	8 (44)
Age, mean (±SD)	59 (±16)	47 (±13)	50 (±17)
BMI, (mean±SD)	26.2 (±5)	25.5 (±5)	NA
RF and/or ACPA, n (%)	36 (78)	NA	NA
Erosions, n (%)	27 (60)	NA	NA
Disease activityPGA, mean (SD)*BASDAI, mean (SD)	3.1 (2.6)NA	3.6 (2.2)30 (19)	NANA
Comorbidities			
Hypertension, n (%)	10 (22)	7 (14)	NA
Diabetes	6 (13)	3 (6)	NA
Obesity	3 (6.5)	2 (4)	NA
MACES or VTE	6 (13)	1 (2)	NA
Treatments			
Corticosteroids (number of exposed patients)	16/46	3/48	0
Mean dose of steroids among exposed patients (SD)	6 mg (4)	5.8 mg (3.8)	0
csDMARDs	MTX (11)	MTX (6)/sulfasalazine (2)	NA
TNFi monotherapy	2	22	NA
TNFi+csDMARD	11	12	NA
RTX monotherapy	1	NA	NA
RTX+csDMARD	10	NA	NA
Abatacept	2	NA	NA
JAKi	3	0	NA
Tocilizumab	5	NA	NA
Anti-IL17	NA	7	NA
SARS-CoV-2 diagnosis			
Clinical	2	5	0
Lung CT	2	1	0
RT-PCR/serology	42	42	18
COVID severity			
Intensive care unit	2†	0	0
Lung >50%	4†	0	0

* PGA assessed by a Visual Analogic Scale ranging from 0 to 10.

† MTX monotherapy.

ACPA, Anti Citrullunated Peptide Antibody; BASDAI, Bath Ankylosing Spondylitis Disease Activity Index; csDMARD, conventional synthetic DMARD; DMARD, disease-modifying antirheumatic drug; JAKi, Janus kinase inhibitor; MACES, major adverse cardiovascular events; MTX, methotrexate; NA, not available; PGA, Patients’ Global Assessment; RA, rheumatoid arthritis; RF, Rheumatoid Factor; RTX, rituximab; SpA, spondyloarthritis; TNFi, tumour necrosis factor inhibitor; VTE, venous thromboembolic events.

### Lack of viral persistence in the blood, nasopharynx and stools under immunomodulatory treatments

No SARS-CoV-2 RNA was detected in the stools and sera in all samples obtained at months 1 and 3. Four patients (2 RA; ABA/RTX treatment; 2 SpA; anti-TNF/anti-IL17 treatment) had positive RT-PCR with very low to low quantification at the 1-month visit (mean Ct 36). None of these 4 patients experienced a severe form of COVID-19.

### Immunological cellular profile of recovered COVID-19 RA and SpA patients

**Figure 1:**
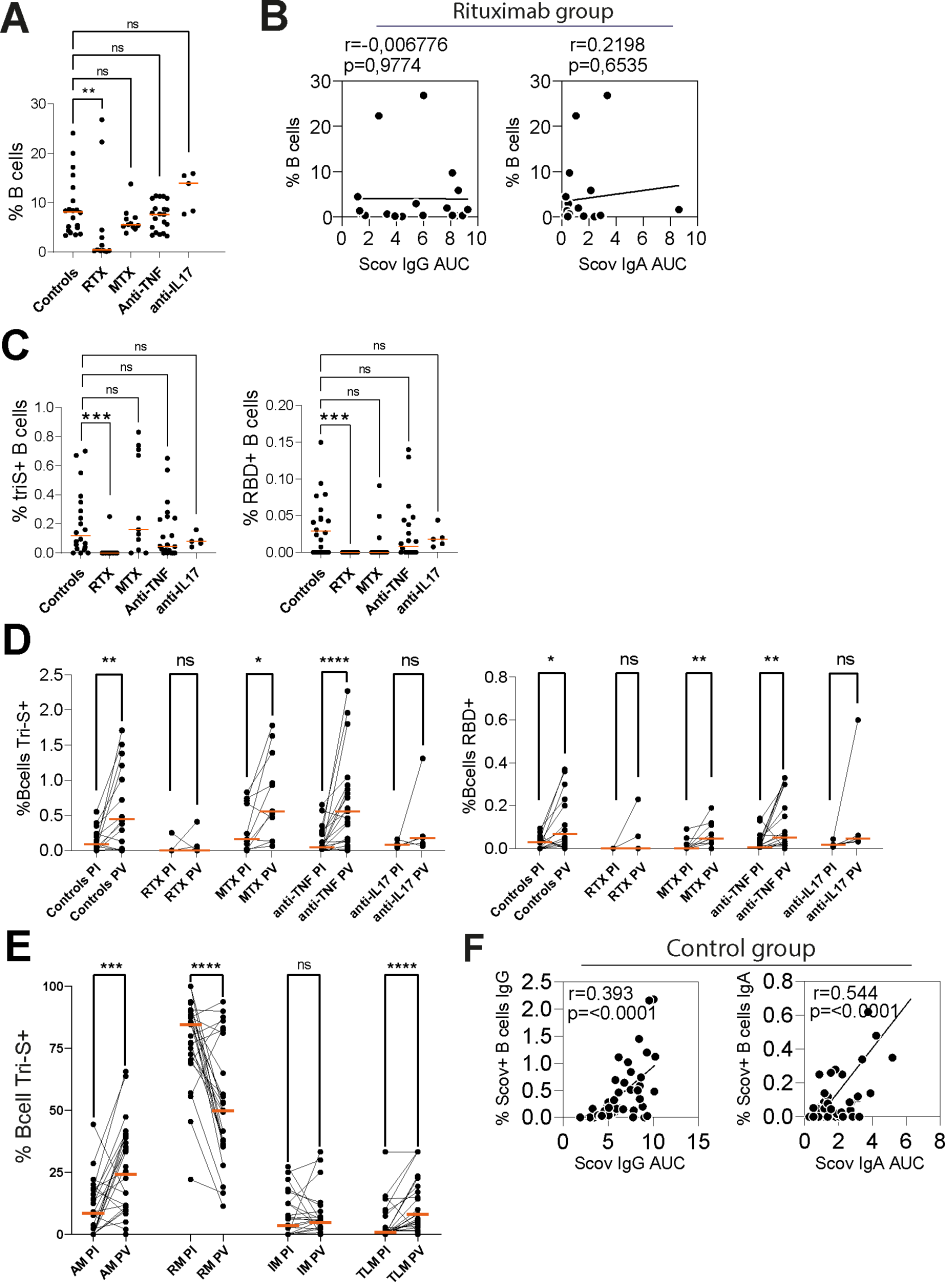
SARS-CoV-2 memory B cells. (**A**) Dot plot showing the percentage of B cells (CD19^+^) in the blood of convalescent COVID-19 individuals treated with different therapies postinfection. Medians are shown; two-sided Mann-Whitney U test, **p<0.01 (**B**) Correlation plots showing the frequency of B cells in blood versus the AUC binding values of serum IgG and IgA against Scov protein from Rituximab treated patients. P values were calculated using two-tailed Pearson correlation test. (**C**) Dot plots showing the frequency of SARS-CoV-2 Scov^+^ and RBD^+^ B cells postinfection. Medians are shown; two-sided Mann-Whitney test, ***p<0.001. (**D**) Before-after dot plot showing the percent of Scov ^+^ and RBD^+^ B cells after infection and vaccination. Medians are shown; two-sided Wilcoxon matched-pairs test, *p<0.05, **p<0.01, ***p<0.001. (**E**) Before-after dot plot showing the distribution of Scov^+^ class-switched memory B-cell subset frequencies from all patients after infection and vaccination. RM (resting memory, CD27^+^CD21^+^), AM (activated memory, CD27^+^CD21^−^), IM (Intermediate memory, CD27^−^CD21^+^), TLM (Tissue-like memory, CD27^−^CD21^−^). Medians are shown; two-sided Wilcoxon matched-pairs test, ***p<0.001, ****p<0.0001. (**F**) Correlation plots showing the frequency of Scov^+^ IgA^+^ or IgG^+^ B cells in blood vs the AUC binding values of serum IgA and IgG antibodies to Scov from the control group after infection and vaccination. P values were calculated using two-tailed Pearson correlation test. AUC, area under the curve; MTX, methotrexate; PI, postinfection; PV, postvaccination; RBD, receptor-binding domain.

We observed several specific changes of immune cell populations, some linked to the underlying treatment or the infection or both. In the MTX-treated patients, slight changes were observed in the T-cell compartments with increased naïve CD4+T cells balanced with decreased CD4^+^ central memory and CD4+effector memory T cells (online supplemental figure S1). Regulatory T cells were also significantly decreased in this treatment group. Follicular helper T cells tended to be decreased. In the anti-TNF-treated group, we observed a slight decrease in αβ T cells and in non-classical monocytes (CD14^−^, CD16^+^) compared with controls. All the other analysed cell populations were comparable to the controls (online supplemental figure S1). The B-cell compartment was preserved, except for patients treated with rituximab (figure 1A). Two patients for whom the last rituximab infusion was performed 7 and 8 months before the COVIRIC inclusion visit showed a high level of peripheral blood memory B cells. Both patients had a combo therapy with MTX (15 and 20 mg/week, respectively) and were under low-dose corticosteroids (5 mg/day and 1 mg/day, respectively). Rituximab-treated patients also had slight modifications of their T-cell compartments including increasedαβ T-cells, a trend towards decreased naïve CD8^+^ T cells, decreased central memory CD8^+^ T cells and increased CD8^+^ TEMRA (Effector Memory expressing CD45RA) (online supplemental figure S1).

### Anti-CD20 treatment impairs IgA and IgG antibody responses against SARS-CoV-2 Spike on infection and vaccination

**Figure 2:**
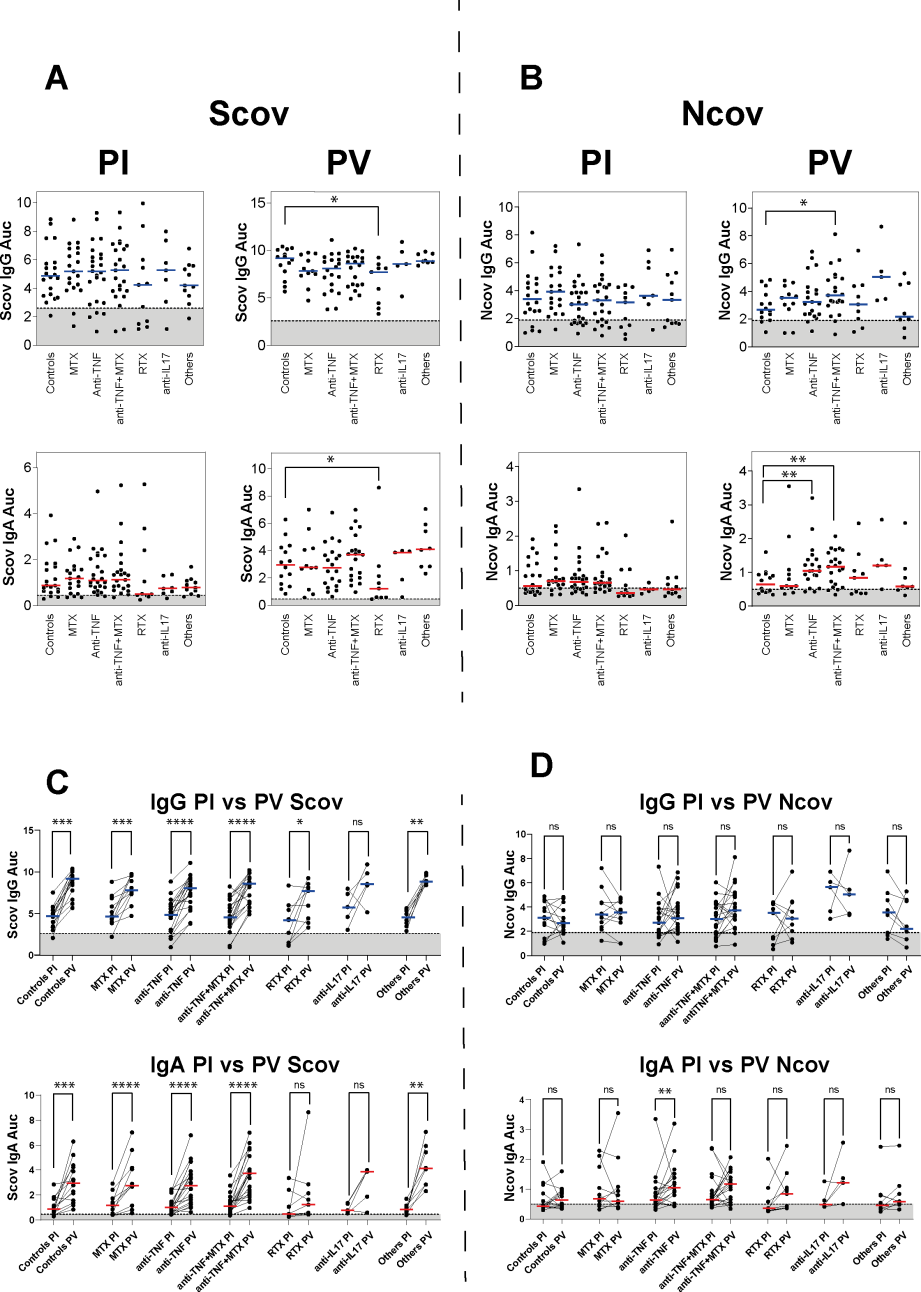
Antibody responses to SARS-CoV-2 postinfection and vaccination. Dot plot showing the IgG and IgA antibody binding to SARS-CoV-2 S (**A**) or N protein (**B**) from SARS-CoV-2 infected (PI) and vaccinated (PV) patients according to different therapies. The values are determined by ELISA with serially-diluted sera and expressed as area under the curve (AUC). The dashed line and shaded region indicate values under the detection threshold determined by receiver operating characteristic (ROC) analyses. Medians are shown; two-sided Mann-Whitney test, *p<0.05, **p<0.01 was considered significant. (**C**) Before-after dot plot showing Scov and (**D**) Ncov antibody responses following infection (PI) and vaccination (PV) for each treatment group. Medians are shown; two-sided Wilcoxon matched-pairs test, **p<0.01, ***p<0.001, ****p<0.0001.

To characterise humoral responses, we first evaluated the IgG and IgA seroreactivity by ELISA of convalescent and vaccinated individuals to soluble recombinant Wuhan SARS-CoV-2 trimeric Spike (Scov) and nucleocapsid protein (Ncov). Antibody responses to SARS-CoV-2 antigens were assessed in all individuals 114±56 days PI and 116.5±92 days PV (online supplemental figure 2A). In order to perform ELISA titrations of IgA and IgG against SCOV and Ncov, it was first necessary to establish cut-off thresholds. These were determined by means of receiver operating characteristic (ROC) analyses, with a trade-off between sensitivity and specificity (see online supplemental figure 2B). Serum Scov-specific and Ncov-specific antibody levels were heterogeneous within each group of treated and non-treated patients after SARS-CoV-2 infection or vaccination (figure 2A, B). Also, anti-Scov and anti-Ncov IgA antibody titres were globally weaker than for IgGs. Patient treated with RTX had lower anti-Scov IgA and IgG antibody levels than the other treated and control groups. However, the difference did not reach statistical significance. Further analysis revealed that the proportion of patients treated with rituximab (RTX) who had undetectable anti-Scov IgG (36.4%; 4 out 11) and IgA (45.4%, 5 out 11) PI was higher compared with other groups (figure 2A). The mean interval between SARS-CoV-2 infection and the last rituximab infusion was 4.5 months (±2.5 months). Nevertheless, no association was observed between patients with impaired SARS-CoV-2 antibody responses and the time of the last rituximab infusion before infection. Regarding PV, patients in the RTX group had statistically lower IgA and IgG responses against Scov compared with the other groups. Of note, anti-Ncov IgA titres were higher PV for patients treated with anti-TNF and anti-TNF+MTX as compared with the control group (p<0.05) (figure 2B).

For each treatment group, vaccination elicited significantly higher anti-Scov IgA and IgG responses, including for patients who had undetectable antibody levels PI (figure 2C). The increase of anti-Scov IgA and IgG antibodies after vaccination for RTX and anti-IL17 treatment groups was weak and not statistically significant, likely due to the limited number of samples analysed. Patients were all vaccinated with Pfizer–BioNTech, Moderna or AstraZeneca vaccines that elicit an immune response only against the Scov protein. Hence, anti-Ncov IgA and IgG antibody levels were not improved after vaccination, except for the group treated with anti-TNF that showed a slightly higher level of anti-Ncov IgA (figure 2D). However, patient data concerning the potential for asymptomatic reinfection with SARS-CoV-2 were not available. To assess the sustainability of systemic antibody responses to Scov and Ncov proteins, IgA and IgG titres were quantified during a longitudinal follow-up after infection and vaccination (online supplemental figure 2C). Serum levels of anti-Scov IgG antibodies slowly declined several months after infection for each treatment group and reached its lowest level at time point 4, corresponding to 370±34 days PI. As previously described, vaccination induced a high anti-Scov IgA and IgG antibody rebound. Circulating levels of anti-Ncov IgA and IgG antibodies remained stable after infection and were not affected by vaccination. In contrast, nasal Scov-specific IgG and IgA responses were undetectable for a majority of patients and controls (85%; n=120) at the time of sampling (average of 120 days PI) (online supplemental figure 2D).

### SARS-CoV-2 antibody response is highly correlated with the percentage of SARS-CoV-2-specific B-cells

To examine the memory B-cell antibody response to SARS-CoV-2 Spike protein, peripheral blood B cells from patients after infection and vaccination were stained with fluorescently-labelled recombinant trimeric S (Scov) and the RBD proteins (online supplemental figure 3A). Anti-CD20 therapy drastically depleted total circulating blood B cells in treated patients (figure 1A). However, B-cell depletion in RTX-treated patients was not associated with serum antibody response to Scov protein since certain patients developed strong antibody responses despite having very low numbers of circulating B cells (figure 1B). Except for patients treated with rituximab, the frequency of Scov-reactive and RBD-reactive B cells was not impacted by treatments (figure 1C). Despite a slightly higher total B-cell frequency months after infection (online supplemental figure 3B), vaccination increased the percentage of SARS-CoV-2 IgA^+^ and IgG^+^ memory B cells specific to Scov and RBD proteins (figure 1D). Few months after infection, Scov^+^ class-switched memory B cells with a resting memory B-cell phenotype (RM, CD19^+^CD27^+^CD21^+^) were predominant compared with those with an activated memory phenotype (AM, CD27^+^CD21^−^) (figure 1E). Nonetheless, and unlike resting memory B cells, the frequency of circulating activated memory B cells significantly increased (ranging from 8% to 24%) following vaccination. In addition, vaccination induced the expansion of tissue-like memory B cells (TLM) characterised by the absence of CD21 and CD27 surface expression. The shift from RM B cells to AM and TLM B cells following vaccination was observed for all treatment groups (online supplemental figure 3C). Importantly, blood Scov^+^ IgA^+^ and IgG^+^ B cell frequencies for the control group were correlated to serum anti-Scov IgA and IgG titres, respectively (figure 1F).

### Decreased percentage of SARS-CoV-2-Spike-specific activated CD8^+^ T cells in RA patients treated with rituximab

**Figure 3:**
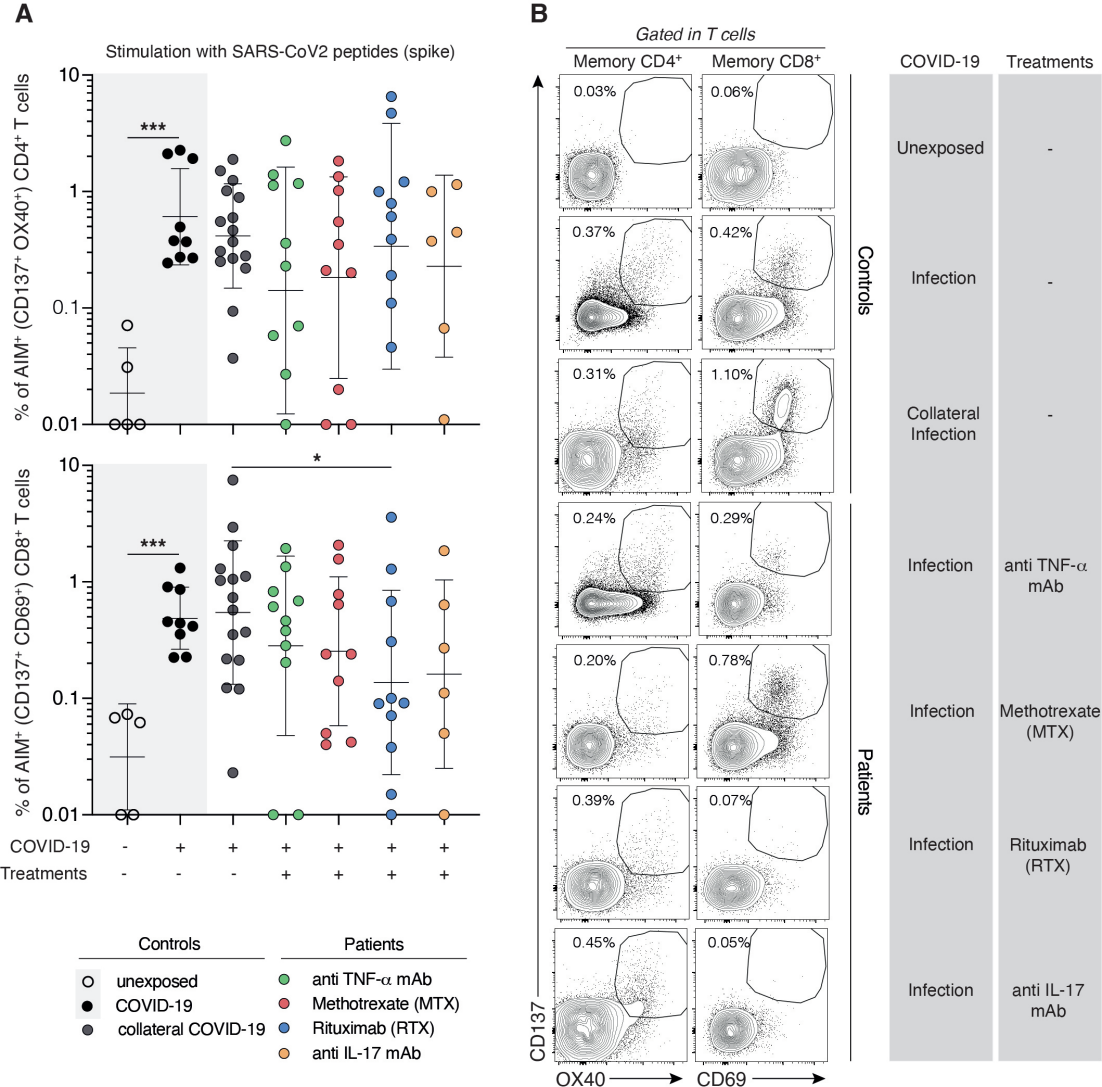
Specific SARS-CoV-2-Spike activated T cells in patients according to treatments. (**A**) Dot plot graphs summarising the frequencies of specific SARS-CoV2-spike AIM+ (CD137^+^OX40^+^) CD4+cells (upper panel) and AIM+ (CD137^+^CD69^+^) CD8+cells from patients and donors collaterally infected with SARS-CoV-2 (grey circle, n=16), patients treated with anti-TNF-a (green circle, n=10), methotrexate (red circle, n=10), rituximab (blue circle, n=10) or anti-17 (yellow circle, n=6). Unexposed (clear circle) and SARS-CoV-2 infected (black circle) internal controls are plotted in grey box (left part of the dot plot). Dashed line represents the positivity threshold of CD4 and CD8 activation with SARS-CoV-2 Spike peptides. Each circle represents a biological sample of independent donors or patients. The horizontal bars represent the median±SD. Data obtained from 10 independent experiments. Group of values were compared two by two using Mann-Whitney tests. *p<0.05; ***p<0.001. (**B**) Representative dot plots of flow cytometry analysis showing CD137, OX40 and CD69 expressions in memory CD4 (left panel) and CD8 (right panel) T cells of donors and patients stimulated with SARS-CoV2 Spike peptides. Data obtained are summarised in A.

**Figure 4:**
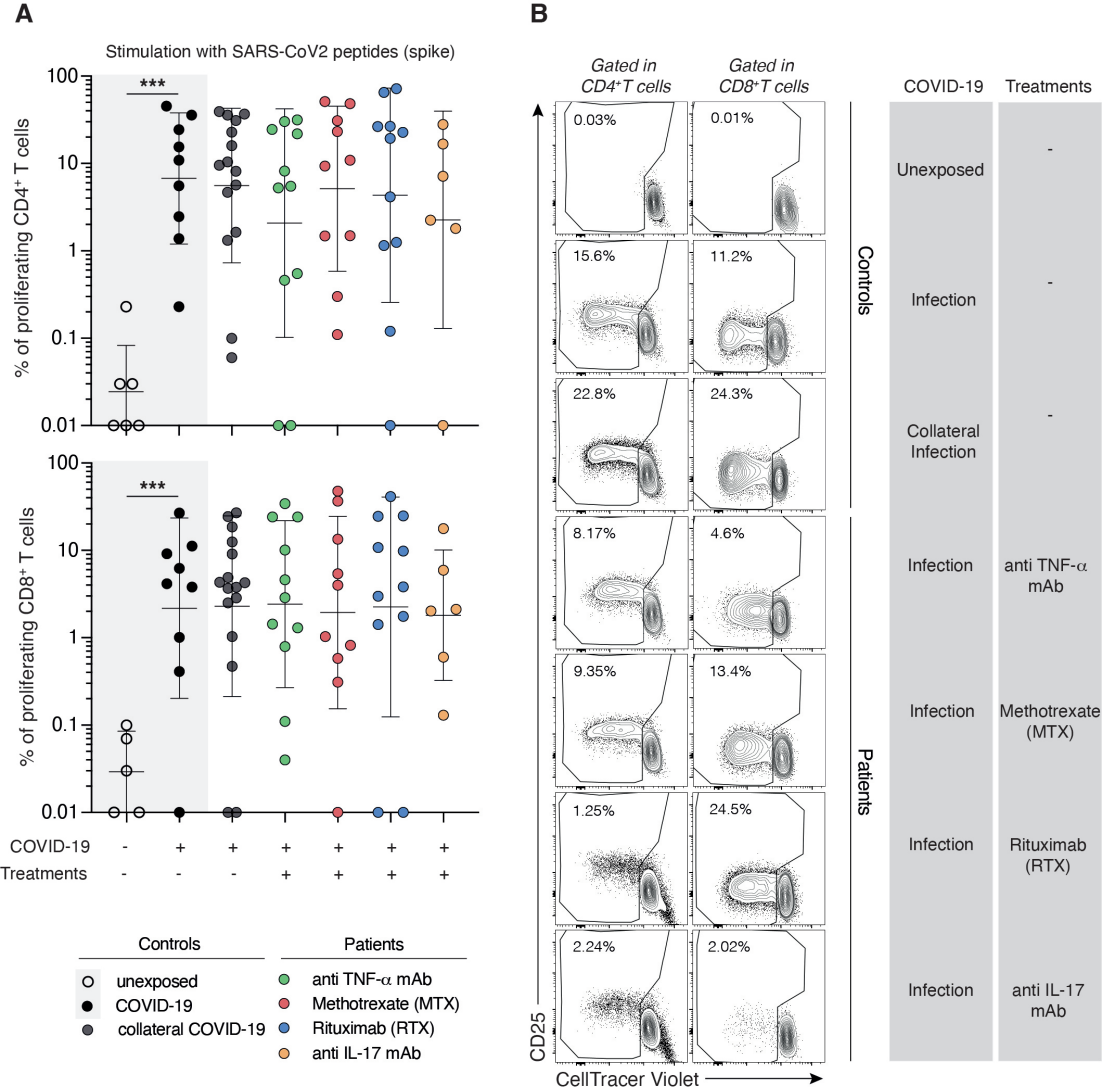
Proliferation of specific SARS-CoV-2-Spike T cells in patients according to their treatments. (**A**) Dot plot graphs summarising the frequencies of specific SARS-CoV2− Spike AIM+ (CD137+OX40+) CD4+cells (upper panel) and AIM+ (CD137+CD69+) CD8+cells from patients and donors collaterally infected with SARS-CoV-2 (grey circle, n=16), patients treated with anti-TNF-a (green circle, n=11), methotrexate (red circle, n=10), rituximab (blue circle, n=10) or anti-17 (yellow circle, n=6). Unexposed (clear circle) and SARS-CoV2 infected (black circle) internal controls are plotted in grey box (left part of the dot plot). Dashed line represents the positivity threshold of CD4 and CD8 proliferation after 5 days of culture with SARS-CoV-2 spike peptides. Each circle represents a biological sample of independent donors or patients. The horizontal bars represent the median±SD. Data obtained from 10 independent experiments. Group of values were compared two by two using Mann-Whitney tests. ***p<0.001. (**B**) Representative dot plots of flow cytometry analysis showing CellTrace Violet (CTV) dilutions in memory CD4 (left panel) and CD8 (right panel) T cells of donors and patients stimulated with SARS-CoV2 Spike peptides during 5 days. Data obtained are summarised in A.

We then studied SARS-CoV-2 specific activated CD4^+^ and CD8^+^ T-cells by T-cell stimulation with Spike peptides. In all treatment groups, the percentage of activated CD4^+^ T (CD137^+^ OX40^+^) cells was comparable to the control group. In contrast, we observed a reduction in SARS-CoV-2-specific activated CD8^+^ T cells (CD137^+^ CD69^+^) exclusively in the rituximab-treated group (figure 3A, B). When considering the percentage of patients under the threshold of activated CD8^+^ T cells, we observed that 63% of patients in the rituximab-treated group had undetectable activated CD8^+^ T cells (compared with 18% in the healthy control group, 18% in the TNFi group, and 33% in the IL17i group). We found no significant association between impaired CD8^+^ T cell activation and either the residual CD19^+^ B cell count at the time of the last rituximab infusion or the absolute CD19+B cell count at the time of CD8+T cell activation markers assessment. Even if both analyses yielded similarly non-significant results, these findings should be interpreted with caution given the limited number of patients studied. Analysis of CD4^+^ and CD8^+^ T-cell proliferation for 5 days in the presence of Spike-derived peptides showed results comparable to the control group for all treatment groups (figure 4A, B). As a positive control for the activation and proliferation capacities of CD4^+^ and CD8^+^ T cells, activation and proliferation assays using a pool of Epstein-Barr virus (EBV)/influenza A virus peptides and anti-CD3/CD28 coated beads were used on the same patients’ and controls’ samples (online supplemental figures 4 and 5). We also considered potential confounding factors. Most patients treated with rituximab who exhibited impaired humoral and CD8^+^ T cell responses were in a state of low RA activity. Comorbidities were evenly distributed between patients with RA and SpA, as well as between those treated with rituximab and those receiving other therapies (p=0.37). As reported in table 1, 12.1% of patients were receiving concomitant corticosteroid therapy. In this context, we found that corticosteroid use was not associated with CD8^+^ T cell activation (table 2, p=1.000). A similar lack of association was observed for CD4^+^ T cell activation (data not shown).

**Table 2:** Parameters associated with SARS-CoV-2 specific CD8+ activation markers

TotalN=55	TCD8 activationN=39	TCD8 no activationN=16	P value
Age	52.0 (14.3)	54.3 (14.6)	46.8 (13.1)	*0.079*
Sex (female)	33 (60.0%)	22 (56.4%)	11 (68.8%)	*0.547*
Disease Control RA SpA	16 (29.1%)18 (32.7%)21 (38.2%)	13 (33.3%)10 (25.6%)16 (41.0%)	3 (18.8%)8 (50.0%)5 (31.3%)	*0.241*
Days of delay between the COVID-19 infection and the visit	118.7 (45.0)	121.3 (50.5)	113.9 (29.5)	*0.584*
Treatment Controls Methotrexate Anti-IL17 Anti-TNF Rituximab Other	16 (29.1%)10 (18.2%)6 (10.9%)11 (20.0%)10 (18.2%)2 (3.6%)	13 (33.3%)7 (17.9%)4 (10.3%)9 (23.1%)4 (10.3%)2 (5.1%)	3 (18.8%)3 (18.8%)2 (12.5%)2 (12.5%)6 (37.5%)0 (0%)	*0.271*
Corticosteroids	7 (12.7%)	5 (12.8%)	2 (12.5%)	*1.000*
Anti-S IgG T2 +/−	49 (89.1%)	38 (97.4%)	11 (68.8%)	***0.006***
Anti-S IgG T2	5.25 (2.21)	5.77 (1.91)	4.20 (2.43)	***0.013***
Anti-N IgG T2 +/−	44 (80.0%)	35 (89.7%)	9 (56.3%)	***0.009***
Anti-N IgG T2	3.66 (1.85)	4.08 (1.84)	2.65 (1.54)	***0.009***

RA, rheumatoid arthritis; SpA, spondyloarthritis.

### Coordination of SARS-CoV-2 IgG antibody and CD8^+^ T cell responses

We next address whether a correlation exists between anti-Scov IgG antibody response and T-cell activation markers, we found a positive and significant correlation between SARS-CoV-2 Scov IgG level and CD8^+^ T cell activation (R=0.4; p=0.0022) but not CD4^+^ T cell activation (R=0.021; p=0.88) (online supplemental figure 6). When considering globally treated patients, those with CD8^+^ T-cell activation markers above the threshold had significantly higher humoral responses assessed by anti-Scov IgG titres (median value 5.5) as compared with those under the threshold (median value 3.9) (p=0.009) (table 2). The same significant association was found between SARS-CoV-2 specific CD8^+^ T-cell activation markers and anti-N IgG antibodies considering either IgG titres (p=0.009) or IgG above the positive threshold (p=0.009) (table 2). A strong correlation was observed between CD4^+^ and CD8^+^ T cell activation markers when including all patients (R=0.44; p=0.0009).

## Discussion

Data on the infection-induced immune responses to SARS-CoV-2 in patients with RA and SpA treated with immunomodulating DMARDs are limited. In contrast, comprehensive information has been gathered on SARS-CoV-2 vaccine responses in these patients.^25^,^33^ Here, we assessed SARS-CoV-2-specific humoral and cellular immune responses in a large cohort of SpA and RA patients treated with DMARDs. We first observed an absence of viral persistence in nasopharyngeal swabs, stools and the blood of controls and DMARDs-treated patients. We confirmed an altered humoral response in rituximab-treated patients with a correlation between the serum antibody response and the percentage of SARS-CoV-2-specific blood B cells. Of major importance, we observed a reduced percentage of SARS-CoV-2-specific CD8^+^ activated T cells in RTX-treated patients, specifically among those with a reduced humoral response to the SARS-CoV-2 Spike protein.

Data on viral persistence in Immune Mediated Inflammatory Diseases patients treated with immunomodulatory therapy are limited. Zollner *et al* reported that SARS-CoV-2 antigens persist in the gut mucosa for months after acute COVID-19 in most patients with inflammatory bowel diseases irrespective of the immunosuppressive therapy or gut inflammation (biopsy tissue through endoscopic assessment 219 days (range, 94–257) after a confirmed COVID-19 infection).^34^ However, SARS-CoV-2 expression was not detectable in stools from those patients at this time point. No data on the medium to long-term viral persistence in RA or SpA patients treated with DMARDs are available to date. The low number of patients with a severe form of COVID-19 in our cohort (n=4) likely explains why we did not observe any viral persistence in the samples analysed in this study.

In response to SARS-CoV-2 infection or vaccination, neutralising antibodies are elicited and target certain epitopes on the Spike protein, particularly the RBD. SARS-CoV-2 convalescent individuals in the healthy control group maintained high serum antiviral IgG and IgA levels several months after infection. SARS-CoV-2 mRNA vaccination increased significantly both SARS-CoV-2 spike-specific serum antibody titres and blood AM B-cell frequencies. Most recovered COVID-19 controls in our study developed both CD4^+^ (87%; OX40^+^ CD137^+^) and CD8^+^ (81%; CD69^+^CD137^+^) T-cell responses against SARS-CoV-2 assessed by Activation-Induced Marker (AIMs), as previously shown by Grifoni *et al*.^35^ In convalescent patients, SARS-CoV-2-specific CD4^+^ T-cell response was correlated with anti-SARS-CoV-2 antibody titres and strongly with SARS-CoV-2-specific CD8^+^ T cell response.^35^

While our study focused primarily on detailed immunological assessments, real-world data have provided complementary insights into vaccine efficacy in patients with chronic inflammatory rheumatic diseases receiving immunosuppressive therapies. Notably, studies from the COVID-19 Global Rheumatology Alliance registry reported reduced vaccine effectiveness in patients treated with B cell-depleting therapies (BCDT). For instance, among 22 fully vaccinated individuals who required hospitalisation for COVID-19, 41% were receiving BCDT, with a high mortality rate of one-third.^36^ Furthermore, multiple studies have confirmed impaired immunogenicity to COVID-19 vaccines in rituximab-treated patients,^37^,^39^ including lower antibody titres and a more rapid decline over time. These findings underscore the need for tailored vaccination strategies in this vulnerable population, who may remain at increased risk for severe COVID-19 despite full vaccination. In our study, among RA and SpA patients undergoing DMARD treatment, only rituximab had a profound impact on the SARS-CoV-2 spike-specific antibody responses, with a higher number of patients falling below the detection threshold. We observed a preservation of the proliferative capacity of SARS-CoV-2-Spike-specific CD4^+^ and CD8^+^ T cells on peptide stimulation, including in rituximab-treated patients. When evaluating the activation status of CD4^+^ and CD8^+^ T cells, treatment groups showed preserved responses against SARS-CoV-2 for both as compared with the control group. Noteworthy, however, patients in the rituximab treatment group displayed a different pattern with only 4 out of 11 patients (36%) demonstrating CD8^+^ T-cell responses as assessed by AIM (CD69^+^CD137^+^) (compared with 82% for healthy controls). Furthermore, the CD8^+^ T-cell response against a pool of EBV/influenzae virus peptides also had the tendency to decrease in the rituximab group, with 54% of rituximab-treated patients not responding in contrast to only 13% in the control group. These observations suggest that rituximab treatment has a significant impact on the activation of virus-specific CD8^+^ T cells during infections. In contrast, activation and proliferation of specific CD4+T cells were preserved.

The results of our study also suggest a narrow link between SARS-CoV-2-specific systemic humoral and CD8^+^ T-cell responses, especially with the observation in the rituximab-treated group in which both were found altered. In the context of autoimmune diseases, B-CD8 crosstalk has already been observed, with B-cell depletion leading to a dampened pathogenic CD8 T-cell response.^40^ For instance, this is the case in antineutrophil cytoplasmic antibody-associated vasculitis (AAV)^41^ or in multiple sclerosis treated with ocrelizumab.^42 43^ Rituximab-treated AAV patients have reduced frequency of CD8^+^CD45RA^+^CCR7^-^ T cells (TEMRA) and a reduced production of cytokine/chemokine by CD8+T cells. In line with this, our immunophenotyping data at the inclusion visit indeed demonstrate a significant decrease of TEMRA CD8^+^ cells in the rituximab group. In the context of antimicrobial immunity, it is well known that patients treated with anti-CD20 BCDT are at higher risk of viral and opportunistic infections, suggesting a combined B-cell/T-cell immunodeficiency.^44^ The mechanisms by which B-cell depletion can affect the expansion of virus-specific CD8^+^ T cells are under investigation and may rely on the cross-presentation of viral antigens by B-cells to CD8 T cells, and/or on the secretion of cytokines by B cells (IL-2, IL-4, IL-15, IL-27, IL-17 and IFN type I).^40^ In mice models, B-cell deficiency is associated with reduced specific CD8^+^ T-cell expansion on influenza virus or Modified Vaccinia virus Ankara infection.^45^ Reciprocally, CD8^+^ T cells can also boost B-cell responses.^46^ A small pool of CD8^+^ T cells differentiate into antigen-specific long-lived memory CD8 T cells. Some of these cells express CXCR5 and locate in the B-cell area of secondary lymphoid tissues.^47^ These CD8^+^CXCR5^+^ T cells have an effector memory phenotype and could provide stimulatory signals to support B-cell survival and associated humoral response. Notably, SARS-CoV-2-specific CD8^+^ T cells generated on vaccination predominantly display an effector memory phenotype.^44^ Hence, it can be inferred that SARS-CoV-2-specific B-cell and CD8^+^ T-cell responses mutually influence each other, leading to the development of robust immune responses. However, it has been observed that patients treated with anti-CD20 therapy are still able to develop strong S-specific CD8^+^ T-cell responses following SARS-CoV-2 mRNA vaccination, despite exhibiting poor systemic humoral responses. This ability, however, may depend on the T-cell-stimulating components of the vaccine used.^48 49^ Several factors likely contribute to the robustness of the CD8^+^ T-cell response to vaccination as well as to the interplay between B cells and CD8+T cells during the vaccinal response (such as the antigenic target, vaccine type, adjuvants and more).

Our study was able to include a substantial number of patients with RA or SpA who were infected with COVID-19, as well as a control group. However, this work has several limitations. First, the control group was not matched on sex, gender and comorbidities with RA and SpA patients. As controls, we selected first-degree relatives residing in the same household as the index cases at the time of their COVID-19 infection and who were themselves infected during the same period. It should first be noted that the mean age of RA and SpA patients is inherently and expectedly different. Ideally, two separate control groups should have been included—one age-matched to the RA patients and another to the SpA patients. Moreover, this control group was particularly challenging to recruit, as not all household members were infected simultaneously, and some were not infected at all. Consequently, it was not feasible to further apply matching criteria based on sex, age or comorbidities. Second, the relatively small sample size in each treatment group has limited the statistical power for some comparisons. For this reason, all comparisons were evaluated using non-parametric tests. Nevertheless, we acknowledge that the study may be underpowered to detect smaller effect sizes and that the results should be interpreted with caution.

In conclusion, this study provides reassuring evidence that the majority of patients with RA or SpA receiving DMARD therapy are capable of mounting a SARS-CoV-2-specific immune response following infection or vaccination. Most patients, including those treated with rituximab, developed a robust CD4+T cell response against the SARS-CoV-2 Spike protein, even after a non-severe infection. However, rituximab was associated with a selective impairment in CD8+T cell activation in response to Spike peptides, alongside diminished humoral responses. This cohort is of particular interest because it actually reflects a context of first exposure to a novel viral agent in the absence of available vaccines. This allowed us to evaluate the natural immune response in immunosuppressed patients, as well as their response to the first-generation SARS-CoV-2 vaccines. In both settings—PI and PV—we observed a marked reduction in anti-SCoV IgA and IgG antibody responses in rituximab-treated patients. Rituximab therapy was also associated with a reduced frequency of SARS-CoV-2-specific activated CD8+T cells following infection. Interestingly, similar findings were observed using EBV and influenza-derived peptides, suggesting a broader impact of rituximab on CD8+T cell responses to viral antigens in general. These findings underline the potential B–CD8+T cell crosstalk and the broader impact of B-cell depletion on antiviral immunity. These observations underscore the relevance of developing vaccines capable of inducing strong CD8+T cell responses, which could be particularly beneficial for individuals receiving immunomodulatory therapies for inflammatory rheumatic diseases—regardless of the specific viral pathogen involved.

## Methods

### Patients and controls

The COVIRIC study is a prospective cohort study including non-vaccinated patients with RA or SpA treated with csDMARDs or b/tsDMARDs and infected with SARS-CoV-2. 96 patients were initially included in the COVIRIC study after a confirmed SARS-CoV-2 infection (at least one of the following elements had to be present: typical clinical symptoms, a positive RT-PCR test result, a positive serology test result and/or a compatible chest CT scan). Two patients with SARS-CoV-2 infection retained exclusively on clinical symptoms (anosmia/ageusia) were further excluded from the initial cohort due to negative humoral response to infection, but positive humoral response to vaccination. In the end, the COVIRIC study was based on the analysis of 94 patients. Patients included in the COVIRIC study were infected between February and November 2020. During this period, the SARS-CoV-2 Spike D614G variant was the widely predominant variant, resulting in a high degree of homogeneity in the following immunological analyses. The first group of controls (n=20) were people living in the same household (spouses, adult children) and infected at the same time as the index patients. Two additional control groups were included in the study, collected at Imagine Institute. The first group consisted of healthy volunteers who had not been exposed to SARS-CoV-2 (N=5), while the second group comprised individuals who were infected with SARS-CoV-2 from 1 to 6 months prior (N=9). Nasal swabs, stool and blood samples were collected at inclusion and viral loads assessed by qRT-PCR. Peripheral blood mononuclear cells (PBMCs) and serum samples were collected at inclusion and at each follow-up visit in order to assess SARS-CoV-2-specific humoral and cellular responses. Immunophenotyping assessing adaptive, innate-like and innate cell populations was exclusively performed at inclusion.

### Cell culture and sample preparation

PBMCs were isolated from heparinised whole blood of patients and healthy donors by Ficoll-Paque density gradient (Lymphoprep, Proteogenix) using standard procedures. PBMCs and serum samples from the patients and controls were frozen at −80°C immediately, and PBMCs were transferred in liquid nitrogen until use. Cell phenotyping and functional assay were performed after thawing of PBMCs in warm Panserin 401 (Pan Biotech) medium supplemented with 5% human AB serum (Bio West), penicillin (100 U/mL), streptomycin (100 µg/mL) and benzonase nuclease (100 U/mL, ThermoFischer Scientific). PBMCs were filtered and resuspended in complete medium at 1×10^7^ cells/mL.

### Expression and purification of viral proteins

Codon-optimised nucleotide fragments encoding stabilised versions of SARS-CoV-2 Spike (HexaPro) (Scov) ectodomains followed by a fold on trimerisation motif and C-terminal tags (Hisx8-tag, Strep-tag and AviTag) were synthesised and cloned into pcDNA3.1/Zeo(+) expression vector (Thermo Fisher Scientific). Synthetic nucleotide fragments coding for Wuhan SARS-CoV-2 RBD and nucleocapsid protein (N) followed by C-terminal tags (Hisx8-tag, Strep-tag, and AviTag) were cloned into pcDNA3.1/Zeo(+) vector. Glycoproteins were produced by transient transfection of exponentially growing Freestyle 293-F suspension cells (Thermo Fisher Scientific, Waltham, Massachusetts, USA) using polyethylenimine (PEI) precipitation method as previously described.^50^ Proteins were purified from culture supernatants by high-performance chromatography using the Ni Sepharose Excel Resin according to manufacturer’s instructions (GE Healthcare), dialysed against PBS using Slide-A-Lyzer dialysis cassettes (Thermo Fisher Scientific), quantified using NanoDrop 2000 instrument (Thermo Fisher Scientific) and controlled for purity by SDS-PAGE using NuPAGE 3%–8% Tris-acetate gels (Life Technologies), as previously described^50^). AviTagged Scov proteins were biotinylated using the BirA biotin-protein ligase bulk reaction kit (Avidity, LLC). SARS-CoV-2 RDB protein was coupled to DyLight 650 using the DyLight Amine-Reactive Dyes kit (Thermo Fisher Scientific).

### Detection of SARS-CoV2 in nasal swabs

Nasal swabs were collected and stored at −80°C, thawed and centrifuged to pellet particulate matter. Spike-specific IgG and IgA responses were assessed in supernatants using ‘S-flow’ as previously described.^51^

### Detection of SARS-CoV-2 in stool samples

Nasal swabs were collected and stored at −80°C. RNA extraction, real-time RT-PCR (rtRT-PCR), and virus isolation and titration were undertaken as previously described.^52^ The viral load in stools was calculated and expressed in number of RNA copies per g of stool.

### Detection of SARS-CoV-2 viral load by droplet-based digital PCR

Plasma samples were extracted using the Cellfree200 V7 DSP 200 protocol with the QIAsymphony DSP virus/pathogen mini kit (QIAGEN, UK). Samples loaded onto the QIAsymphony SP as instructed by the manufacturer, with a 200 µL sample input volume (composed of 150 µL of sample and 50 µL of NaCl) and 60 µL elution output volume of AVE buffer, unless stated (QIAGEN, UK). The samples were then stored at −80°C until the droplet digital PCR (ddPCR) step. SARS-CoV-2 RT-ddPCR assays were performed using the One-Step RT-ddPCR Advanced Kit for 90 Probes (Bio-Rad Laboratories, Hercules, California, USA) and the QX200 ddPCR platform (Biorad). A 2-plex RT-ddPCR assay was developed, which targets the Nucleocapside (N1) gene of the SARS-CoV-2 positive-strand RNA genome with specific FAM-probe and primers Cy5-labelled probe for the detection of a human housekeeping gene (RNAseP). RNAseP positivity was necessary to validate the RT-PCR assay prior to any further analysis. Briefly, 9.9 µL of extracted RNA was diluted in a 22 µL final reaction volume containing 5.5 µL of One Step SuperMix, 2.2 µL of Reverse Transcriptase, 1.1 µL of 300 mM DTT (One-Step RT-ddPCR Advanced Kit for Probes, Bio-Rad), 1.1 μL of primers and probes mix (final probe concentration: 200 nM each, final primer concentration: 600 nM each) and 2.2 µL (QS) of nuclease-free water. Then, each sample was primarily partitioned into 13 000–20 000 droplets using the QX200 Droplet Generator (Bio-Rad). PCR amplification was then performed on a C1000 Touch thermal cycler (Bio-Rad). The droplet reading and quantification were performed using the QX200 Droplet Reader and data analysis was performed using the QuantaSoft Analysis Pro software (Bio-Rad).

### Analysis of SARS-CoV-2-specific humoral responses

All human sera were heat-inactivated at 56°C for 60 min and SARS-CoV-2-specific ELISAs were performed as previously described (86, 92). Briefly, high-binding 96-well ELISA plates (Costar, Corning) were coated overnight with 125 ng/well of purified recombinant SARS-CoV-2 S or N proteins. After washings with 0.1% Tween 20-PBS (washing buffer), plates were blocked for 2 hours with 3% milk, 5% sucrose, 1% Tween 20-PBS (Blocking buffer), washed and incubated with serially diluted human sera. Total sera were diluted 1:100 followed by 7 consecutive 1:3 dilutions in PBS-BSA 1%. After washings, the plates were revealed by incubation for 1 hour with goat HRP-conjugated anti-human IgG or anti-human IgA antibodies (Jackson ImmunoResearch, 0.8 µg/mL final) and by adding 100 µL of HRP chromogenic substrate (ABTS solution, Euromedex) after washing steps. Optical densities were measured at 405 nm (OD405nm). Experiments were performed using HydroSpeed microplate washer and Sunrise microplate absorbance reader (Tecan Männedorf, Switzerland).

### B-cell immunophenotyping

PBMCs were isolated from donors’ blood using Ficoll Plaque Plus (GE Healthcare). For B-cell phenotyping, biotinylated Scov were incubated for 30 min at 4°C with streptavidin R-phycoerythrin (PE) conjugate (Invitrogen, Thermo Fisher Scientific) and streptavidin PE-Cyanin7 (PE-Cy7, BD Pharmingen). Then B cells were incubated for 30 min at 4°C with biotinylated Scov-coupled to PE and Cy7 and SARS-CoV-2 RDB coupled to DyLight 650, washed once with 1% FBS-PBS (FACS buffer), and incubated for 30 min at 4°C with a cocktail of mouse anti-human antibodies: CD19 Alexa 700 (HIB19, BD Biosciences, San Jose, California, USA), CD21 Brilliant violet (BV) 421 (B-ly4, BD Biosciences), CD27 PE-CF594 (M-T271, BD Biosciences), IgG BV786 (G18-145, BD Biosciences), IgA FITC (IS11-8E10, Miltenyi Biotec, Bergisch Gladbach, Germany). To exclude dead cells, CD19+B cells were stained using LIVE/DEAD aqua fixable dead cell stain kit (Molecular Probes; Thermo Fisher Scientific). Cells were then washed and fixed in 4% paraformaldehyde-PBS. Flow cytometric analyses of stained cells were performed using a CytoFLEX instrument (Beckman Coulter), and the FlowJo software (V.10.6, FlowJo).

### T-cell immunophenotyping

Ex vivo phenotypic analyses or cell staining after PBMC stimulation were performed according to standard flow cytometry methods. The gating strategy for the flow cytometry-based analysis of T-cell subsets is presented in online supplemental figure 7. The staining of surface antigens was carried out for 30 min in the dark at 4°C. The following monoclonal antibodies conjugated to fluorescein isothiocyanate (FITC), R-PE, PE-Cy7, Peridinin-chlorophyll-cyanin5.5 (PerCP-Cy5.5), allophycocyanin (APC), allophycocyanin-Cyanin7 (APC-Cy7), alexa-700, BV421, BV510, BV605, BV711, BV650 or BV785 were used in optimal titrated concentrations: anti-CD3 (SK7), anti-CD4 (RPTA-T4), anti-CD8 (SK1), anti-CD14 (63D3), anti-CD16 (3G8), anti-CD25 (BC96), anti-CD27 (O323), anti-CD45RA (HI100), anti-CD45RO (UCHL1), anti-CD56 (QA17A16), anti-CD57 (QA17A04), anti-CD70 (113-16), anti-CD137 (4B4-1), anti-CD161 (HP-3G10), anti-CD183 (G025H7), anti-CD185 (J252D4), anti-CD196 (G034E3), anti-CD197 (G043H7) and anti-CD279 (EH12.EH7), anti-TCRab (IP26), anti-HLA-Dr (LN3), anti-KLRG1 (SA231A2) all purchased from BioLegend. To exclude dead cells, Zombie NIR fixable viability (BioLegend) was added for the last 10 min of incubation. iNKT cells were detected by staining with anti-Va24-Ja18 (6B11-BioLegend) and anti-Vb11 (C21- Beckman Coulter). MAIT cells were detected by staining with anti-Va7.2 (3C10- BioLegend) and anti-CD161 (HP-3G10 -BioLegend). All data were collected on the LSR-Fortessa cytometer (BD Biosciences), and instrument performance was monitored daily with Rainbow Calibration Particles (BD Biosciences).

### AIM assay

Control and patient PBMCs were cultured in Panserin 401 (Pan Biotech) medium supplemented with 5% heat inactivated human AB serum (Bio West), penicillin (100 U/mL) and streptomycin (100 µg/mL) at 1×10^6^ cells/well in 96-well U-bottom (Corning) with the relevant peptides (1 µg/mL, PepMix SARS-CoV-2 or EF peptide Pool, JPT) in the presence of 1 µg/mL of soluble anti-CD28 antibody (CD28.2, ebioscience) during 24 hours of incubation at 37°C, 5% CO2. The PepMix SARS-CoV-2 pool (JPT) contains 315 peptides derived from a peptide scan (15mers with 11 aa overlap) through Spike glycoprotein. The EF pool (JPT) is a positive control pool of 20 peptides selected from EBV and Influenza A virus defined T-cell epitopes. Stimulation controls were performed with equal concentrations of DMSO in PBS (unstimulated) or with anti-CD3+CD28 coated beads (Invitrogen) as positive control, respectively. The stimulation was stopped by incubation in 20 mM EDTA for 5 min and Activation Induced Cell Markers surface expression (4-1BB (CD137), CD69, OX-40 (CD134)) was analysed in memory CD4 and CD8 T cells by flow cytometry.

### Proliferation assay

Control and patients PBMCs were cultured for 5 days in complete medium with DMSO, PepMix SARS-CoV-2 peptides (1 µg/mL, JPT), EF peptide Pool (1 µg/mL, JPT) or anti-CD3+CD28 coated beads (Invitrogen) with human recombinant IL-2 (10 IU/mL, PeproTech). Cell proliferation was monitored by labelling cells with the violet dye (Violet Proliferation Dye 450, BD Biosciences) prior to stimulation. After 5 days of culture, cells were harvested and Violet dye dilution was assessed by flow cytometry.

### Data analysis and statistics

In stimulation experiments, frequencies of activated and proliferating CD4 or CD8 T cells were background-subtracted, with the frequency of unstimulated samples representing the background. The threshold of activation and proliferation of T cells was determined using the following calculation: Mean of unstimulated sample frequencies+2×SD of unstimulated sample frequencies. A non-parametric test was used to compare cell frequency between groups of samples (Mann-Whitney U*-*test with two-tailed distribution). Flow cytometry data were analysed using FlowJo V.10.8.0 software (Tree Star). Mann-Whitney rank test was used to compare unpaired population and Wilcoxon test to compare matched-pairs population. GraphPad Prism software (V.8.2, GraphPad Prism) was used for plotting and statistical analysis. A p<0.05 is considered significant. Principal component analysis was performed using the prcomp function in R Studio Server (V.1.4.1103).

## Supplementary material

10.1136/rmdopen-2025-005673 online supplemental file 1

## Data Availability

Data are available on reasonable request.
