# Supplementary material for: Adaptive immune responses to SARS-CoV-2 in DMARD-treated patients with chronic inflammatory rheumatisms
Source: RMD Open. 2025 Jul 5;11(3):e005673. doi: 10.1136/rmdopen-2025-005673 (PMC12228473; doi:10.1136/rmdopen-2025-005673)
Supplement: online supplemental file 1 [file rmdopen-11-3-s001.docx]

**Supplementary Figures**

**Adaptative immune responses to SARS-CoV-2 in DMARDs-treated patients with chronic inflammatory rheumatisms – The COVIRIC study**

Maxime Beretta^1§^, Emmanuel Martin^2,3§^, Olivier Fogel^4§^, Clementina Lopez-Medina^5^, Cyril Planchais^1^, Thomas Bruneau^6^, Pedro Goncalves^7^, Jérôme Avouac^4^, Francis Berenbaum^8^, Jérémie Sellam^8^, Bruno Fautrel^9,10^, Jacques Morel^11^, Béatrice Parfait^12^, James P. Di Santo^7^, Sylvie Behillil^13^, Sylvie van der Werf^13^, Hélène Péré^6^, Sylvain Latour^2,3*^, Hugo Mouquet^1*^, Corinne Miceli Richard^3,4,14*^

**FIGURE S1**


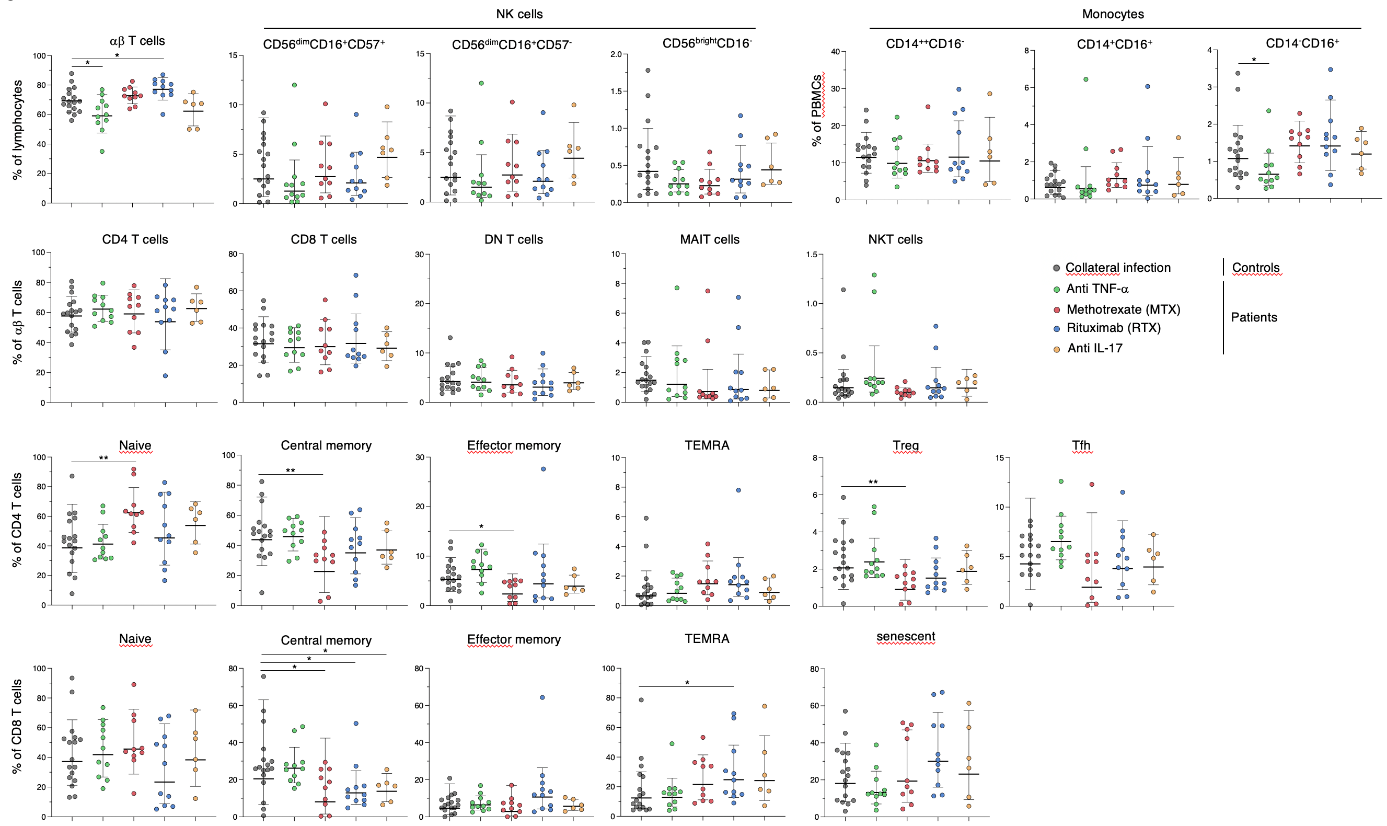


**FIGURE S2**

^
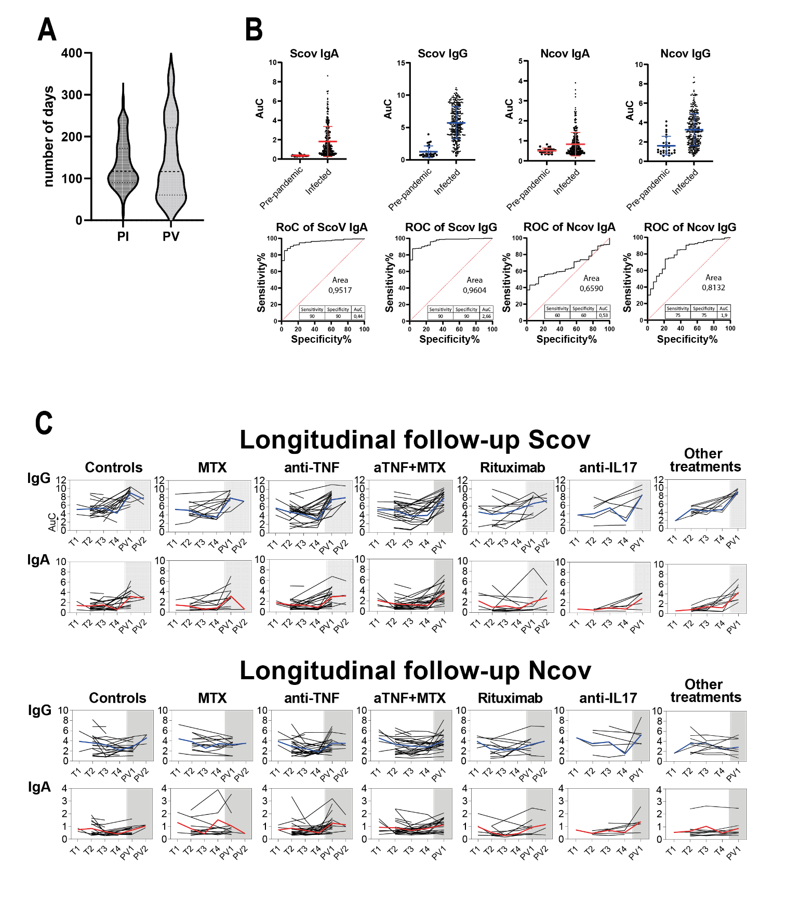
^


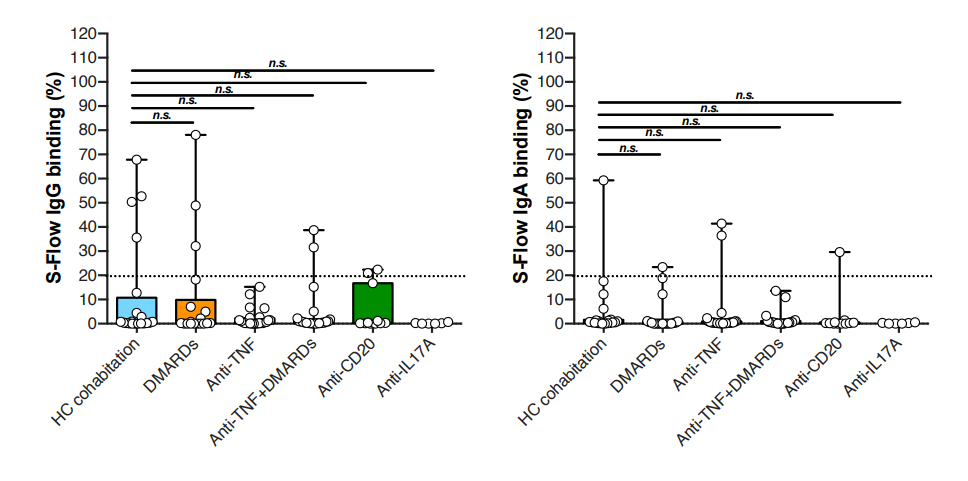


**D**

**FIGURE S3**


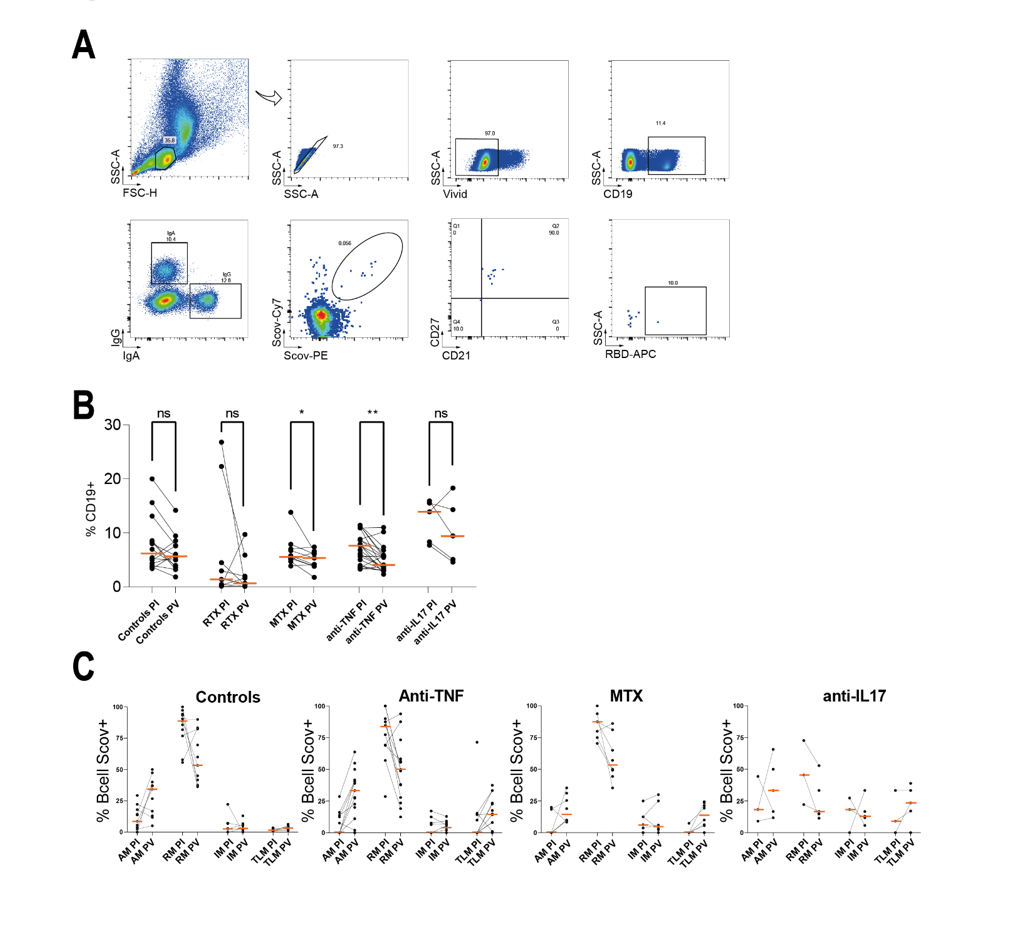


**FIGURE S4**


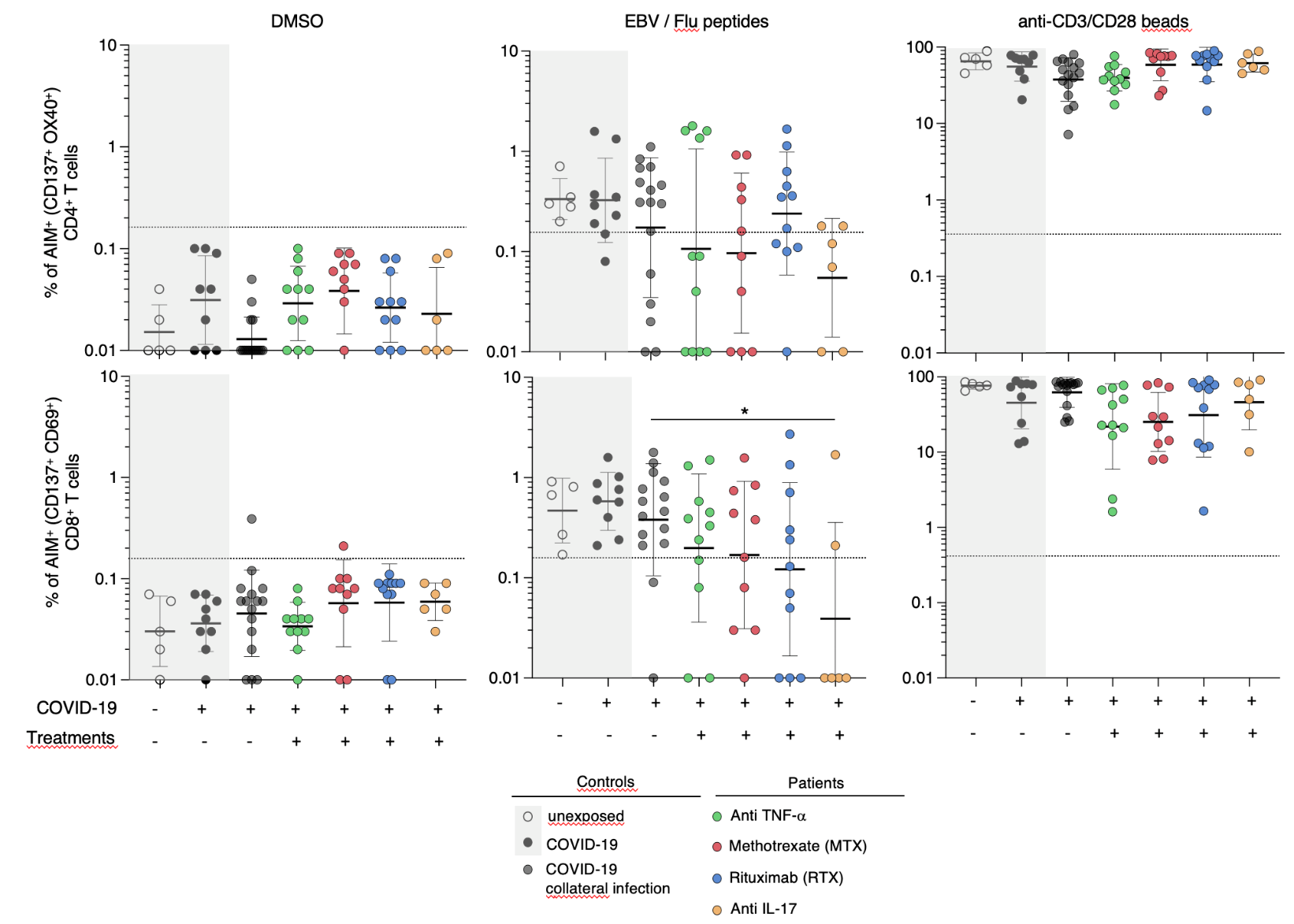


**FIGURE S5**


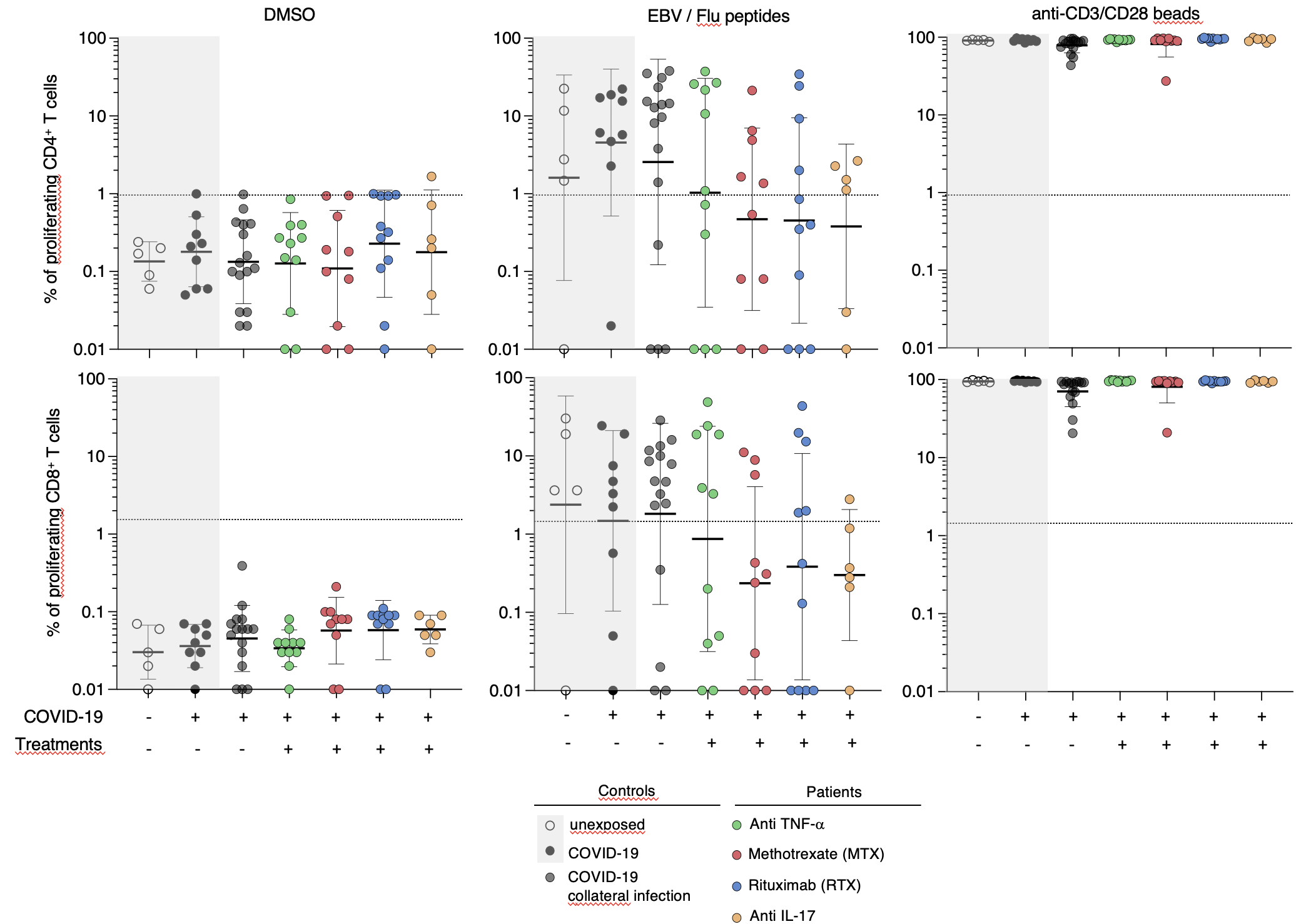


**FIGURE S6**

**FIGURE S7**

**Supplementary Figure Legends**

**Figure S1. Immunophenotyping of SARS-CoV-2 infected donors and rheumatoid arthritis (RA) and spondyloarthritis (SpA) patients.**

**(A)** Frequencies of αβ T cells, NK and monocytes subpopulations in lymphocytes and PBMCs, respectively, are represented in the dot plot graphs. **(B)** Frequencies of CD4+, CD8+, CD4-CD8-, innate like T cells (MAIT and iNKT) gated in CD3+ T cells are depicted in dot plot graphs. **(C)** Frequencies of naïve (CD31+CD45RA+CCR7-), central memory (CD45RA-CCR7+CD27-), effector memory (CD45RA-CCR7+CD27-) and exhausted effector memory/TEMRA (CD45RA+CCR7+CD27-) compartments, follicular helper (Tfh, CD45RA-CCR7+CD127+CD25-CXCR5+) and regulatory T cells (Treg, CD45RA-CCR7lowCD25+) in CD4+ cells are represented in the dot plot graphs. **(D)** Frequencies of naïve (CD31+CD45RA+CCR7-), central memory (CD45RA-CCR7+CD27-), effector memory (CD45RA-CCR7+CD27-), exhausted effector memory/TEMRA (CD45RA+CCR7+CD27-) compartments, senescent cells (CD8+CD57+) in CD4+ cells are shown in the dot plot graphs. **(A-D)** All data were obtained from FACS analysis after cell-specific staining. Grey dots correspond to healthy family members infected with SAR-CoV-2 (infection occurring at the same time and place) (n=17). The infected patients with rheumatoid arthritis are symbolized according to their treatment: green dot (anti TNF-a, n=11), red dots (Methotrexate, n=10), blue dots (Rituximab, n=10) and yellow dots (anti IL-12/23+anti IL-17, n=6). Each circle represents an independent biological sample of donor or patient. The horizontal bars represent the median±sd. Data obtained from height independent experiments. Group of values were compared two by two using Mann-Whitney tests. *, P < 0.05; **, P < 0.01; ***, P < 0.001; ****, P < 0.0001.

**Figure S2. (A)** Violin plot showing sampling time deviation after infection (PI) or vaccination (PV) in days. **(B)** ROC graphs comparing Scov and Ncov ELISA IgA and IgG seroreactivity between SARS-CoV-2 infected individuals and pre-pandemic controls. AUC cutoff values corresponding to a trade-off between sensitivity and specificity are indicated. **(C)** Curves showing IgG and IgA antibody binding to Scov and Ncov as AUC values over time after infection (T1, T2, T3 and T4) or after vaccination (PV1 et PV2). Blue and Red lines indicate respectively IgG and IgA average curves for each group. **(D)** Curves showing IgG and IgA antibody binding to SCov in nasal swabs according to the different treatment groups.

**Figure S3. (A)** Flow-cytometric cytograms showing the gating strategy used to analyze cellular humoral response to fluorescently labeled Scov and RBD. SSC, side scatter; FSC, forward scatter. **(B)** Before-after dot plot showing the frequency of circulating B cells after infection and vaccination according to treatments. Medians are shown; two-sided Wilcoxon matched-pairs test, *P < 0.05, **P < 0.01. **(C)** SARS-CoV-2 Scov^+^ class-switched memory B cells showing a resting memory (RM, CD27^+^CD21^+^), activated memory (AM, CD27^+^CD21^-^), intermediate memory (IM, CD27^-^CD21^+^) or tissue-like memory phenotype (TLM, CD27^-^CD21^-^) after infection and vaccination separated according to treatments.

**Figure S4. Antigenic or anti CD3/28 activation of T cells in RA patients according to treatments.**

Same as in the Figure 2A except that plotted AIM+ cells frequencies correspond to memory CD4 (upper panel) or CD8 (upper panel) of donors and patients stimulated or not (left panel) with peptides of Epstein Barr virus +Influenza A virus (middle panel) or anti CD3/CD28 coated beads (right panels) during 24 hours.

**Figure S5. Antigenic or anti CD3/28 proliferation of T cells in RA patients according to treatments.**

Same as in the Figure 3A except that plotted proliferating cells frequencies correspond to memory CD4 (upper panel) or CD8 (upper panel) of donors and patients stimulated or not (left panel) with Epstein Barr virus + Influenza A virus peptides (middle panel) or anti CD3/CD28 coated beads (right panels) during 24 hours.

**Figure S6: Correlation between SARS-CoV-2 specific CD4+ T cells, SARS-CoV-2 specific CD8+ T cells and SARS-CoV-2 antibodies.**

1. Correlation between SARS-CoV-2 Spike-specific CD4+ T cells and SARS-CoV-2 Spike-specific CD8+ T cells (AIM positive cells)
2. Correlation between SARS-CoV-2 Spike-specific CD4+ T cells and anti-Spike IgG (titre)
3. Correlation between SARS-CoV-2 Spike-specific CD8+ T cells and anti-Spike IgG (titre)

**Figure S7 - Gating strategy for the analysis of activated SARS-CoV2 spike specific T cells.**

Representative FACS dot plot illustrating the PBMC gating strategy used to identify activated SARS-CoV-2 spike-specific CD4+ and CD8+ T cells in a SARS-CoV-2-infected donor. This analysis was performed 24 hours after PBMC stimulation with pools of SARS-CoV2 spike peptides, EBV/flu peptides or anti CD3/CD28 antibody coated beads. Activated SARS-CoV-2-specific CD4+ and CD8+ memory T cells were detected using a combination of antibodies against CD3, CD8, CD4, CD45RA, CCR7, CD137, OX40, and CD69, supplied by BioLegend. Dead cells were excluded with DAPI staining. Red arrows indicate the gated populations to detect the activated (AIM+: CD137+/OX40+ or CD137+/CD69+) SARS-CoV-2-specific CD4+ and CD8+ memory T cells.
